# Sand supplementation favors tropical seagrass *Thalassia hemprichii* in eutrophic bay: implications for seagrass restoration and management

**DOI:** 10.1186/s12870-022-03647-0

**Published:** 2022-06-16

**Authors:** Zhijian Jiang, Songlin Liu, Lijun Cui, Jialu He, Yang Fang, Chanaka Premarathne, Linglan Li, Yunchao Wu, Xiaoping Huang, Manoj Kumar

**Affiliations:** 1 Key Laboratory of Tropical Marine Bio-resources and Ecology, South China Sea Institute of Oceanology, Chinese Academy of Sciences, Guangzhou, 510301 PR China; 2 Southern Marine Science and Engineering Guangdong Laboratory, Guangzhou, 511458 PR China; 3 Innovation Academy of South China Sea Ecology and Environmental Engineering, Chinese Academy of Sciences, Guangzhou, 510301 PR China; 4 University of Chinese Academy of Sciences, Beijing, 100049 PR China; 5 Sanya National Marine Ecosystem Research Station, South China Sea Institute of Oceanology, Chinese Academy of Sciences, Sanya, 572000 China; 6 Key Laboratory of Tropical Marine Biotechnology of Hainan Province, Sanya Institute of Oceanology, South China Sea Institute of Oceanology, Chinese Academy of Sciences, Sanya, 572000 China; 7 Climate Change Cluster, Faculty of Science, University of Technology Sydney, Sydney, NSW 2007 Australia

**Keywords:** Eutrophic sediment, Sandy sediment, Seagrass, Photosynthesis, Metabolomics, Stable sulfur isotope

## Abstract

**Background:**

Sediment is crucial for the unique marine angiosperm seagrass growth and successful restoration. Sediment modification induced by eutrophication also exacerbates seagrass decline and reduces plantation and transplantation survival rates. However, we lack information regarding the influence of sediment on seagrass photosynthesis and the metabolics, especially regarding the key secondary metabolic flavone. Meanwhile, sulfation of flavonoids in seagrass may mitigate sulfide intrusion, but limited evidence is available.

**Results:**

We cultured the seagrass *Thalassia hemprichii* under controlled laboratory conditions in three sediment types by combining different ratios of in-situ eutrophic sediment and coarse beach sand. We examined the effects of beach sand mixed with natural eutrophic sediments on seagrass using photobiology, metabolomics and isotope labelling approaches. Seagrasses grown in eutrophic sediments mixed with beach sand exhibited significantly higher photosynthetic activity, with a larger relative maximum electron transport rate and minimum saturating irradiance. Simultaneously, considerably greater belowground amino acid and flavonoid concentrations were observed to counteract anoxic stress in eutrophic sediments without mixed beach sand. This led to more positive belowground stable sulfur isotope ratios in eutrophic sediments with a lower Eh.

**Conclusions:**

These results indicated that coarse beach sand indirectly enhanced photosynthesis in *T. hemprichii* by reducing sulfide intrusion with lower amino acid and flavonoid concentrations. This could explain why *T. hemprichii* often grows better on coarse sand substrates. Therefore, it is imperative to consider adding beach sand to sediments to improve the environmental conditions for seagrass and restore seagrass in eutrophic ecosystems.

**Supplementary Information:**

The online version contains supplementary material available at 10.1186/s12870-022-03647-0.

## Introduction

Seagrasses are marine ecosystem engineers that provide important ecological services including nutrient uptake, carbon sequestration, food and habitat for marine animals, and shoreline and sediment stabilization [1–3]. However, global climate change and sustained pressures from coastal development, including dredging and eutrophication (nutrient enrichment), have weakened the capacity of seagrass meadows to support coastal productivity [4, 5]. Eutrophication affects the structure of primary producers in seagrass beds [6–8], modifying sediment origin, grain size and nutrient availability [7, 9]. An increase in sediment clay and silt fractions and high organic matter content might lead to anoxic conditions [7]. Sediment anoxia inhibits respiration and other metabolic functions in seagrass roots, resulting in reduced photosynthesis, leaf number, and the shoot-to-root ratio [10–12]. Meanwhile, sediment nutrient toxicity might induce an imbalanced carbon-nitrogen ratio due to increased carbon demand [13]. Elevated nutrient levels, respiration, and anoxic conditions also enhance sediment sulphide concentrations [14]. This causes sulphide intrusion in seagrasses, as assessed by stable sulphur isotope signals, leading to adverse effects [14]. Sulphide intruding into seagrass interferes with cytochromes in the electron transport chain, leading to a negative energy balance, which eventually results in seagrass mortality [10, 15]. To date, physiological indicators have largely failed to monitor seagrass health and prevent its decline [16]. The alarming decline highlights the urgent need to implement effective seagrass management strategies to prevent habitat decline [17].

Recently, omics-based systems biology (transcriptomics, proteomics and metabolomics) has emerged as a new frontier in seagrass research and has deepened our understanding of their stress tolerance mechanisms and accurately identified biomarkers of their phenotypic plasticity to environmental stress [18, 19]. Metabolomics has been instrumental in connecting the genotype and phenotype of vascular plants under adverse environmental conditions, and has been applied in seagrass research [19–21] providing new insights into diverse cellular pathways to identify stress tolerance biomarkers. Much is known about the effect of environmental stress on the primary metabolites of seagrass [22, 23] and the total content of key secondary metabolites [24]. However, little is known about the response of key secondary metabolite compositions by applying targeted metabolomics techniques.

The seagrass *Thalassia hemprichii* is a dominant tropical species, growing mainly in sandy sediment or coral substrate [25, 26]. Over the past decade, nutrient inputs into seagrass beds in Xincun Bay, Hainan Island, South China Sea, have increased immensely, leading to high eutrophication [4]. Cage farming and shrimp pond cultures produce large quantities of food debris, which modifies the sediment particle sizes [4]. The sediment particle size decreased from coarse to fine. Hypoxic conditions in sediments occur frequently, and the emergence of red tides has been observed in these areas [27]. Overall, these adverse environmental conditions have induced a decline in seagrass beds, resulting in an approximately 50 ha loss [28]. Interestingly, according to our continuous observations, *T. hemprichii* occurrence in Xincun Bay has declined dramatically, especially in the high intertidal zones. Moreover, we observed a relatively low success rate in transplanting and restoring *T. hemprichii* in this bay (personal observation). This failure might be attributed to the desiccation exposure during low tide and sediment composition (mud vs. sand) [29]. Nevertheless, limited studies have considered the effect of sediment type on seagrass physiology [22], especially flavonoids, which are the key secondary metabolites. Flavonoids have been implicated in plant resistance to many stress factors [30]. The ecological plant strategy theory indicates that stressed plants containing high levels of protective flavonoids tend to show low levels of constitutive productivity [30, 31]. Sulfation of flavonoids in seagrass might also mitigate the sulphide intrusion [32], but limited evidence is available.

Therefore, it is imperative to investigate the effect of sediment type on the physiological responses of the dominant tropical seagrass, *T. hemprichii*. We performed a laboratory manipulative experiment by growing *T. hemprichii* under three sediment types (by combining different ratios of in-situ eutrophic sediment and coarse beach sand) and assessed its growth performance by evaluating photosynthetic performance, flavonoid and amino acid profiling, and stable sulfur isotope and elemental composition analysis. Measurements of seagrass photosynthesis, nitrogen and amino acids contents were used to evaluate the plant growth, whereas measurements of δ^34^S and flavonoids were used to assess the extent of sulphide intrusion in seagrass and the role of flavonoids in mitigating sulphide intrusion, respectively. We examined leaf fluorescence parameters to assess the continuous photosynthetic characteristics of seagrasses in the same leaf in a non-destructive manner [33, 34], without disturbing sediments. The results obtained in this study provide new insights that will aid in understanding the mechanisms controlling seagrass physiological responses to sediment types. This information is critical for strengthening knowledge on improving the success rate of seagrass planting and transplantation in eutrophic coastal areas or in the process of eutrophication and to implementing effective seagrass management strategies to prevent their decline.

## Results

### Sediment physiochemical parameters

The Eh in the sediment type of 1:0, 1:1 and 1:2 were − 177.0 ± 29.4 mV, − 148.7 ± 24.2 mV and − 53.3 ± 17.1 mV, respectively, and the corresponding sediment sulphur contents were (0.020 ± 0.001)%, (0.011 ± 0.001)%, and (0.006 ± 0.002)%, respectively (Table 1). Meanwhile, sediment organic matter also exhibited a decreasing trend with increasing sediment particle size.

**Table 1:** Sediment physiochemical parameters at the end of the experiment

Sediment type	Eh (mV)	S (%)	Organic matter (%)
1:0	−177.0 ± 29.4^a^	0.020 ± 0.001^c^	1.14 ± 0.09^b^
1:1	−148.7 ± 24.2^a^	0.011 ± 0.001^b^	0.75 ± 0.22^a^
1:2	−53.3 ± 17.1^b^	0.006 ± 0.002^a^	0.58 ± 0.04^a^

The different lower case letters indicated significant differences among treatments

### Photosynthesis

**Fig. 1:**
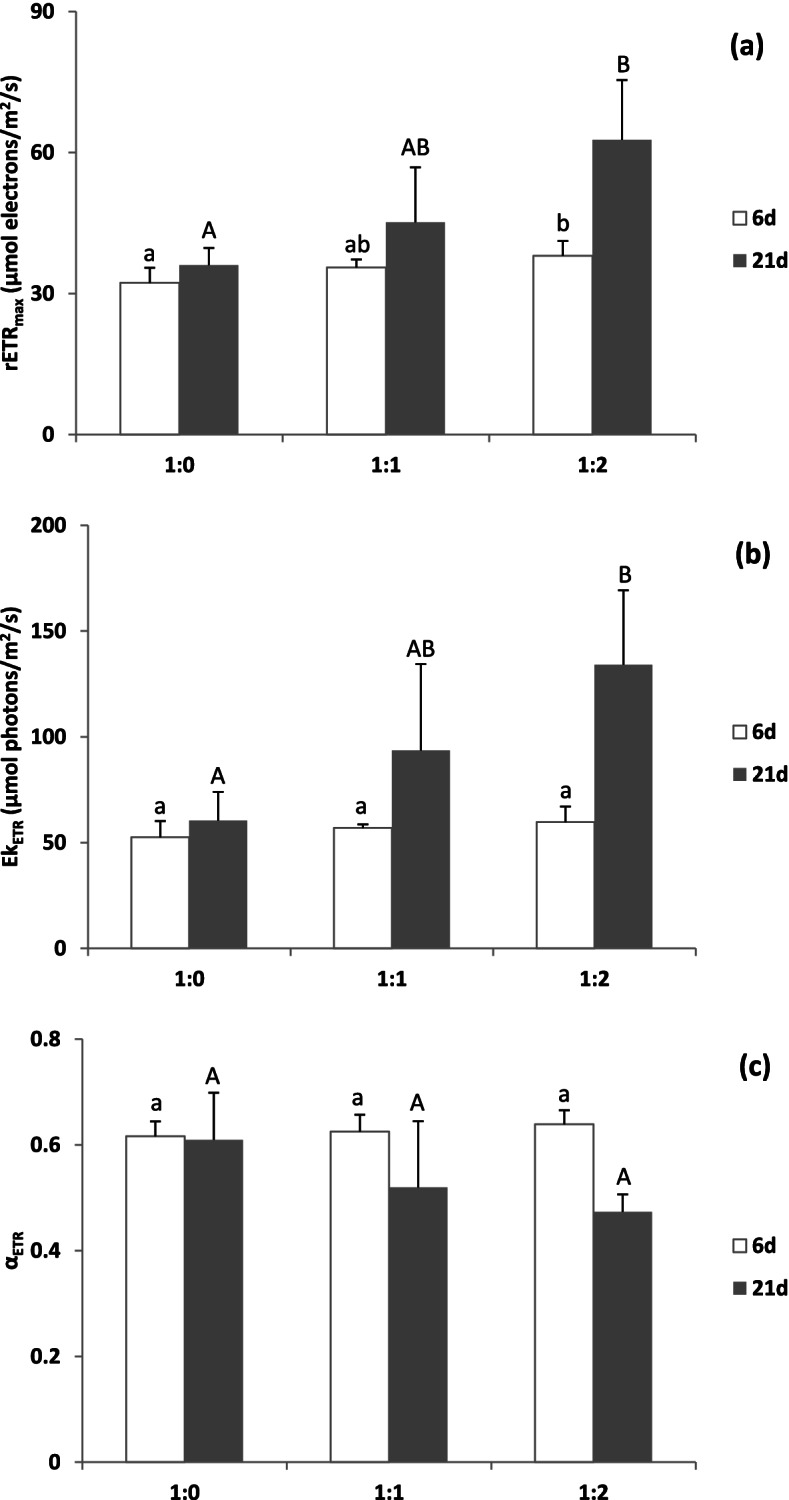
Photosynthetic parameters of *Thalassia hemprichii* including rETR_max_ (the relative maximum electron transport rate, **a**), Ek_ETR_ (the minimum saturating irradiance, **b**) and α_ETR_ (the initial slope of the light limited relationship, **c**) derived from rapid light curve cultured in different sediment types. The lowercase and uppercase letters indicate significant difference at day 6 and day 21, respectively (*P* < 0.05). 1:0, the in-situ sediment without combining with coarse beach sand was added in the tank; 1:1, the combination of half in-situ sediment and half coarse beach sand was added in the tank; 1:2, the combination of 1/3 in-situ sediment and 2/3 coarse beach sand was added in the tank

The effects of sediment type on photosynthetic parameters at the two stages were depicted in Fig. 1. No significant difference was observed in Y (II) (effective quantum yield) on days 6 and 21 (at the end of the experiment) (Additional file 1). Indeed, the differences among treatments on day 6 were not significant, considering the relative maximum electron transport rate (rETR_max_), minimum saturating irradiance (Ek_ETR_) and initial slope of the light-limited relationship (α_ETR_). rETR_max_ and Ek_ETR_ were slightly higher in sediments with larger particle size. However, markedly difference was found at day 21 for both rETR_max_ and Ek_ETR_, with much higher values in the sediment with larger particle sizes (Fig. 1).

### Seagrass nitrogen and compositions of amino acids and flavonoids

**Fig. 2:**
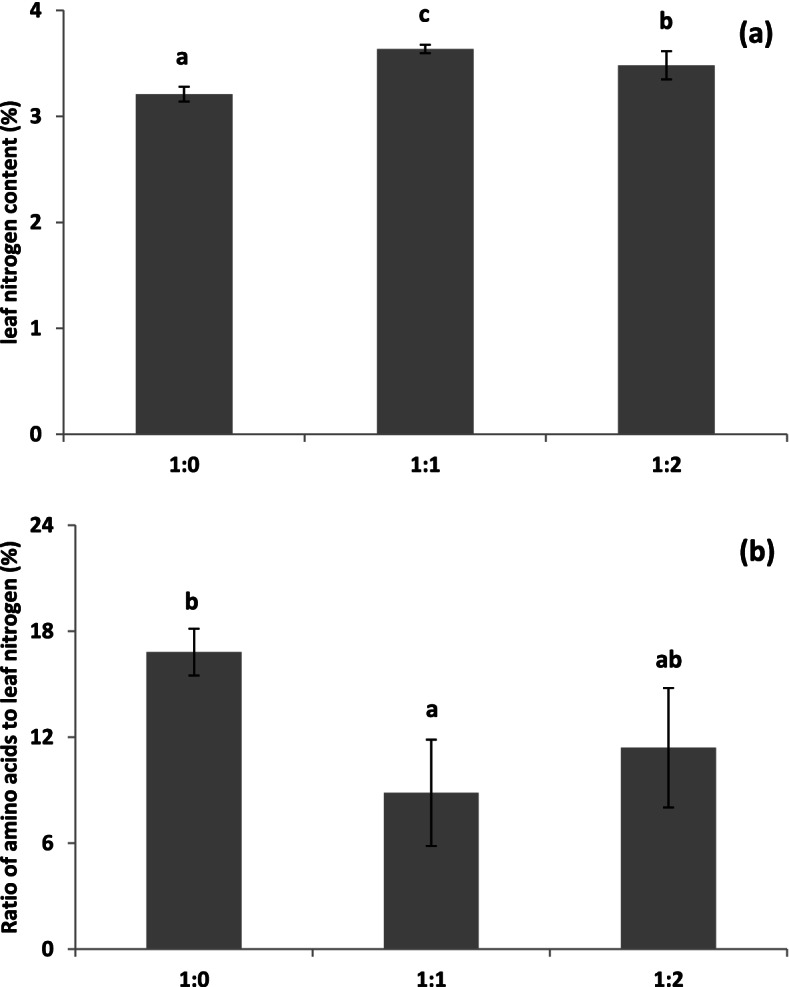
Effect of sediment type on leaf nitrogen (**a**) and the ratio of amino acids to nitrogen (**b**) in seagrass *Thalassia hemprichii*. Different letters on column indicate significant difference (*P* < 0.05). 1:0, the in-situ sediment without combining with coarse beach sand was added in the tank; 1:1, the combination of half in-situ sediment and half coarse beach sand was added in the tank; 1:2, the combination of 1/3 in-situ sediment and 2/3 coarse beach sand was added in the tank

Among the amino acids, proline, sarcosine and lysine were the three main components in the aboveground tissue of *T. hemprichii*, whereas sarcosine, proline and asparagic acid were the three main components in the belowground tissue. The amino acid content in the aboveground tissue was lower than that in the belowground tissue in the sediment 1:0 type, whereas similar concentrations were observed between aboveground and belowground tissue for *T. hemprichii* in both 1:1 and 1:2 sediment types. Significant effects were observed for 11 of the 20 amino acids in the aboveground tissue, whereas effects were observed in 18 amino acids in belowground tissue. Amino acid contents in both above- and belowground tissues in the 1:0 treatment were significantly higher than those in the 1:1 and 1:2 treatments. Sarcosine, proline and alanine in both above- and belowground tissues also showed the same trend. The nitrogen content in the aboveground tissue was significantly higher in the larger sediment particle sizes, whereas the ratio of amino acids to nitrogen in the same tissue showed a contrasting trend (Fig. 2, Table 2, and Table 4).

**Table 2:** Effects of sediment type on the amino acids in the aboveground and belowground tissues of seagrass *Thalassia hemprichii*. 1:0, the in-situ sediment without combining with coarse beach sand was added in the tank; 1:1, the combination of half in-situ sediment and half coarse beach sand was added in the tank; 1:2, the combination of 1/3 in-situ sediment and 2/3 coarse beach sand was added in the tank

Amino acid	Aboveground tissue						Belowground tissue					
	1:0		1:1		1:2		1:0		1:1		1:2	
	content (μg/g)	ratio (%)	content (μg/g)	ratio (%)	content (μg/g)	ratio (%)	content (μg/g)	ratio (%)	content (μg/g)	ratio (%)	content (μg/g)	ratio (%)
glycine	38.2 ± 5.6^a^	0.71	17.9 ± 4.2^b^	0.56	21.4 ± 5.9^b^	0.54	125.8 ± 22.5^A^	1.32	27.7 ± 4.1^B^	0.80	36.1 ± 1.9^B^	0.92
sarcosine	927.1 ± 194.8^a^	17.18	182.8 ± 98.5^b^	5.69	457.9 ± 286.4^b^	11.57	2393.1 ± 618.4^A^	25.15	305.0 ± 38.6^B^	8.83	511.8 ± 254.0^B^	13.07
alanine	296.4 ± 40.0^a^	5.49	74.7 ± 31.4^b^	2.33	155.0 ± 100.4^b^	3.91	605.9 ± 120.9^A^	6.37	103.0 ± 7.3^B^	2.98	149.2 ± 45.9^B^	3.81
valine	93.2 ± 17.9^a^	1.73	27.9 ± 13.9^b^	0.87	51.0 ± 21.1^b^	1.29	147.2 ± 9.0^A^	1.55	31.4 ± 1.7^B^	0.91	35.4 ± 10.3^B^	0.90
proline	1234.0 ± 54.6^a^	22.87	860.8 ± 267.9^a^	26.81	979.6 ± 201.1^a^	24.74	1532.4 ± 225.5^A^	16.11	711.5 ± 44.9^C^	20.60	930.2 ± 65.2^B^	23.75
threonine	55.5 ± 4.0^a^	1.03	37.3 ± 14.4^a^	1.16	39.0 ± 13.8^a^	0.98	288.4 ± 39.8^A^	3.03	92.6 ± 10.8^B^	2.68	65.3 ± 5.1^C^	1.67
isoleucine	41.9 ± 9.5^a^	0.78	11.6 ± 6.2^b^	0.36	22.8 ± 8.2^b^	0.58	62.6 ± 11.3^A^	0.66	12.2 ± 1.5^B^	0.35	13.2 ± 3.2^B^	0.34
leucine	32.9 ± 6.9^a^	0.61	5.5 ± 2.1^b^	0.17	15.3 ± 9.6^b^	0.39	25.3 ± 5.3^A^	0.27	4.8 ± 1.0^B^	0.14	6.2 ± 0.8^B^	0.16
ornithine	59.4 ± 4.1^a^	1.10	53.5 ± 15.5^a^	1.67	52.2 ± 6.6^a^	1.32	208.6 ± 17.0^A^	2.19	115.8 ± 8.1^B^	3.35	99.6 ± 19.5^B^	2.54
methionine	2.5 ± 0.5^a^	0.05	1.2 ± 0.1^b^	0.04	1.2 ± 0.1^b^	0.03	12.7 ± 0.9^A^	0.13	2.1 ± 0.3^B^	0.06	1.8 ± 0.4^B^	0.05
histidine	20.3 ± 5.4^a^	0.38	12.4 ± 1.1^a^	0.39	21.1 ± 5.8^a^	0.53	49.8 ± 5.1^A^	0.52	13.3 ± 2.3^B^	0.39	11.4 ± 1.8^B^	0.29
phenylalanine	23.6 ± 5.9^a^	0.44	6.8 ± 2.0^b^	0.21	12.9 ± 7.6^ab^	0.33	13.4 ± 4.4^A^	0.14	3.7 ± 0.9^B^	0.11	3.8 ± 0.7^B^	0.10
arginine	14.6 ± 1.7^a^	0.27	6.7 ± 1.1^b^	0.21	9.7 ± 4.5^ab^	0.25	116.9 ± 74.2^A^	1.23	221.7 ± 27.2^B^	6.42	329.8 ± 27.7^B^	8.42
tyrosine	16.5 ± 3.1^a^	0.31	5.9 ± 1.8^b^	0.19	10.5 ± 4.3^ab^	0.27	20.1 ± 2.5^A^	0.21	4.0 ± 1.3^B^	0.12	3.8 ± 0.6^B^	0.10
asparagic acid	350.5 ± 6.2^a^	6.50	339.5 ± 92.3^a^	10.57	299.4 ± 16.4^a^	7.56	1224.5 ± 86.9^A^	12.87	729.9 ± 49.4^B^	21.14	618.1 ± 117.4^B^	15.78
tryptophan	409.0 ± 23.2^a^	7.58	350.4 ± 114.9^a^	10.91	378.1 ± 47.6^a^	9.55	443.7 ± 192.7^A^	4.66	229.4 ± 24.7^AB^	6.64	200.0 ± 60.3^B^	5.11
4-aminobutyric acid	555.5 ± 57.8^a^	10.29	182.0 ± 98.9^b^	5.67	334.2 ± 206.1^ab^	8.44	792.5 ± 112.1^A^	8.33	162.9 ± 47.3^B^	4.72	287.5 ± 51.2^B^	7.34
serine	73.7 ± 4.9^a^	1.36	47.1 ± 8.7^b^	1.47	69.6 ± 16.8^ab^	1.76	176.9 ± 40.1^A^	1.86	50.6 ± 3.0^B^	1.47	51.8 ± 3.0^B^	1.32
lysine	643.5 ± 50.1^a^	11.92	574.8 ± 195.1^a^	17.90	577.8 ± 119.7^a^	14.59	717.3 ± 417.3^A^	7.54	316.5 ± 35.3^A^	9.17	285.2 ± 96.1^A^	7.28
glutamate	508.3 ± 30.6^a^	9.42	412.6 ± 103.2^a^	12.85	450.7 ± 56.1^a^	11.38	557.6 ± 213.6^A^	5.86	315.0 ± 33.4^AB^	9.12	277.1 ± 77.2^B^	7.07
sum	5396.7 ± 378.0^a^	100	3211.5 ± 1064.1^b^	100	3959.5 ± 1108.6^ab^	100	9514.5 ± 1890.8^A^	100	3453.3 ± 247.6^B^	100	3917.3 ± 145.0^B^	100

The different lower case and upper case letters indicated significant differences for aboveground and belowground tissues among treatments, respectively

Among the flavonoids, galuteolin, luteolin and isoquercitrin were the three most abundant components in the aboveground tissues of *T. hemprichii* in the three sediment types. For belowground tissue, catechin, isoquercitrin, and epicatechin were the three major components in the sediment 1:0 type, whereas catechin, isoquercitrin and luteolin were the leading three components in the sediment 1:1 and 1:2 types. Flavonoid concentrations in the aboveground tissue were lower than those in the belowground tissue in sediment 1:0 and 1:1 types, whereas similar concentrations were observed between above- and belowground tissue for *T. hemprichii* in the 1:2 sediment type. Flavonoids in both above- and belowground tissues were higher in sediment type of 1:0 than in 1:1 and 1:2 sediment types (Table 3 and Table 4).

**Table 3:** Effect of sediment type on flavonoids in the aboveground and belowground tissues of seagrass *Thalassia hemprichii*. 1:0, the in-situ sediment without combining with coarse beach sand was added in the tank; 1:1, the combination of half in-situ sediment and half coarse beach sand was added in the tank; 1:2, the combination of 1/3 in-situ sediment and 2/3 coarse beach sand was added in the tank

Flavonoids	Aboveground tissue						Belowground tissue					
	1:0		1:1		1:2		1:0		1:1		1:2	
	content (μg/g)	ratio (%)	content (μg/g)	ratio (%)	content (μg/g)	ratio (%)	content (μg/g)	ratio (%)	content (μg/g)	ratio (%)	content (μg/g)	ratio (%)
catechin	0.0223 ± 0.0065^a^	0.55	0.0090 ± 0.0041^b^	0.33	0.0115 ± 0.0033^b^	0.43	14.4433 ± 5.4480^A^	67.56	1.1228 ± 0.0646^B^	31.20	0.2845 ± 0.0281^C^	10.20
epicatechin	nd		nd		nd		1.2517 ± 0.1782^A^	5.85	0.0437 ± 0.0016^B^	1.22	nd^C^	
taxifolin	nd		0.0068	0.25	nd		0.0219 ± 0.0101^A^	0.10	0.0146 ± 0.0096^A^	0.41	0.0234 ± 0.0119^A^	0.84
galuteolin	1.9969 ± 0.6946^a^	49.97	1.5255 ± 0.9832^a^	55.46	1.6700 ± 1.0298^a^	62.55	0.0033 ± 0.0013^A^	0.02	0.0069 ± 0.0037^A^	0.19	0.0078 ± 0.0029^A^	0.28
rutin	0.0049 ± 0.0041^a^	0.12	0.0024 ± 0.0007^a^	0.09	0.0026 ± 0.0009^a^	0.1	0.0116 ± 0.0057^A^	0.05	0.0025 ± 0.0001^B^	0.07	0.0101 ± 0.0063^AB^	0.36
isoquercitrin	0.4959 ± 0.2699^a^	12.41	0.1903 ± 0.1332^a^	6.94	0.2901 ± 0.1066^a^	10.87	5.2404 ± 1.1379^A^	24.51	1.4558 ± 0.4994^B^	40.45	2.3086 ± 1.1878^B^	82.76
astragalin	nd		nd		nd		0.0135 ± 0.0016^A^	0.06	0.0102 ± 0.0011^B^	0.28	0.0119 ± 0.0016^AB^	0.43
diosmin	0.0027 ± 0.0006^a^	0.07	0.0040 ± 0.0008^a^	0.15	0.0034 ± 0.0007^a^	0.13	Nd		nd		nd	
quercetin	0.0439 ± 0.0166^a^	1.10	0.0076 ± 0.0061^b^	0.28	0.0165 ± 0.0047^b^	0.62	0.3283 ± 0.0829^A^	1.54	0.0544 ± 0.0068^B^	1.51	0.0282 ± 0.0110^C^	1.01
naringenin	0.0175 ± 0.0095^a^	0.44	0.0087 ± 0.0021^a^	0.32	0.0067 ± 0.0018^a^	0.25	0.0073 ± 0.0015^A^	0.03	0.0187 ± 0.0073^B^	0.52	0.0076 ± 0.0035^A^	0.27
luteolin	1.3468 ± 0.2366^a^	33.70	0.9275 ± 0.4247^ab^	33.83	0.6227 ± 0.2648^b^	23.33	0.0581 ± 0.0246^A^	0.27	0.8522 ± 0.6017^B^	23.67	0.1065 ± 0.0668^A^	3.82
apigenin	0.0628 ± 0.0223^a^	1.57	0.0631 ± 0.0118^a^	2.30	0.0443 ± 0.0137^a^	1.66	0.0005 ± 0.000^A^	0.01	0.0153 ± 0.0131^C^	0.43	0.0008 ± 0.0001^B^	0.03
chrysin	0.0013 ± 0.0003^a^	0.03	0.0006 ± 0.0001^b^	0.02	0.0008 ± 0.0001^b^	0.03	Nd		0.0018 ± 0.0007	0.05	nd	
kaempferide	0.0015 ± 0.0007^a^	0.04	0.0009 ± 0.0001^a^	0.03	0.0007 ± 0.0002^a^	0.03	Nd		nd		nd	
sum	3.9956 ± 1.1751^a^	100	2.7421 ± 1.5258^a^	100	2.6691 ± 1.4033^a^	100	21.3797 ± 6.4628^A^	100	3.5973 ± 1.1740^B^	100	2.7894 ± 1.2502^B^	100

*nd* Not detectable (i.e. below the limit of detection); the different lower case and upper case letters indicated significant differences for aboveground and belowground tissues among treatments, respectively

**Table 4:** Statistical analysis for the effects of sediment type on the parameters of *Thalassia hemprichii*. There were two stages for photosynthetic parameters. *P* < 0.05 (significant); *P* < 0.01 (highly significant)

Variable	df	F	*P*	Variable	df	F	*P*
Day 6				Day 21			
Y (II)	2	1.412	0.314	Y (II)	2	1.727	0.256
rETR_max_	2	3.346	0.106	rETR_max_	2	5.312	< 0.05
Ek_ETR_	2	1.020	0.415	Ek_ETR_	2	3.965	0.080
α_ETR_	2	0.466	0.649	α_ETR_	2	1.740	0.254
leaf nitrogen	2	62.547	< 0.01	δ^34^S	2	629.078	< 0.01
ratio of amino acid to leaf nitrogen	2	6.708	< 0.05	Organic matter	2	13.158	< 0.01
Eh	2	21.653	< 0.01	S	2	59.828	< 0.01
aboveground tissue				belowground tissue			
glycine	2	12.702	< 0.01	glycine	2	92.525	< 0.01
sarcosine	2	9.832	< 0.05	sarcosine	2	23.737	< 0.01
alanine	2	8.964	< 0.05	alanine	2	41.459	< 0.01
valine	2	10.335	< 0.05	valine	2	204.800	< 0.01
proline	2	2.841	0.136	proline	2	41.100	< 0.01
threonine	2	2.181	0.194	threonine	2	134.373	< 0.01
isoleucine	2	10.712	< 0.05	isoleucine	2	73.448	< 0.01
leucine	2	12.082	< 0.01	leucine	2	66.719	< 0.01
ornithine	2	0.435	0.666	ornithine	2	42.395	< 0.01
methionie	2	21.875	< 0.01	methionie	2	306.695	< 0.01
histidine	2	3.281	0.109	histidine	2	122.650	< 0.01
phenylalanine	2	6.683	< 0.05	phenylalanine	2	19.644	< 0.01
arginine	2	5.777	< 0.05	arginine	2	14.548	< 0.01
tyrosine	2	8.073	< 0.05	tyrosine	2	94.401	< 0.01
asparagic acid	2	0.737	0.517	asparagic acid	2	39.413	< 0.01
tryptophan	2	0.484	0.639	tryptophan	2	3.826	0.085
4-aminobutyric acid	2	5.708	< 0.05	4-aminobutyric acid	2	57.394	< 0.01
serine	2	4.827	0.056	serine	2	117.694	< 0.01
lysine	2	0.247	0.789	lysine	2	2.830	0.136
glutamate	2	1.419	0.313	glutamate	2	3.955	< 0.080
total amino acids	2	4.433	0.066	total amino acids	2	76.191	< 0.01
catechin	2	6.456	< 0.05	catechin	2	19.131	< 0.01
epicatechin	2	–	–	epicatechin	2	143.001	< 0.01
taxifolin	2	–	–	taxifolin	2	0.590	0.584
galuteolin	2	0.209	0.817	galuteolin	2	2.146	0.198
rutin	2	0.942	0.441	rutin	2	3.498	0.098
isoquercitrin	2	2.143	0.198	isoquercitrin	2	12.003	< 0.01
astragalin	2	–	–	astragalin	2	4.119	0.075
diosmin	2	2.616	0.152	diosmin	2	–	–
quercetin	2	9.660	< 0.05	quercetin	2	35.336	< 0.01
naringenin	2	3.003	0.125	naringenin	2	5.609	< 0.05
luteolin	2	3.882	0.083	luteolin	2	4.859	0.056
apigenin	2	1.279	0.345	apigenin	2	23.055	< 0.01
chrysin	2	8.173	< 0.05	chrysin	2	–	–
kaempferide	2	3.753	0.088	kaempferide	2	–	–
total flavonoids	2	0.883	0.461	total flavonoids	2	26.375	< 0.01

**Fig. 3:**
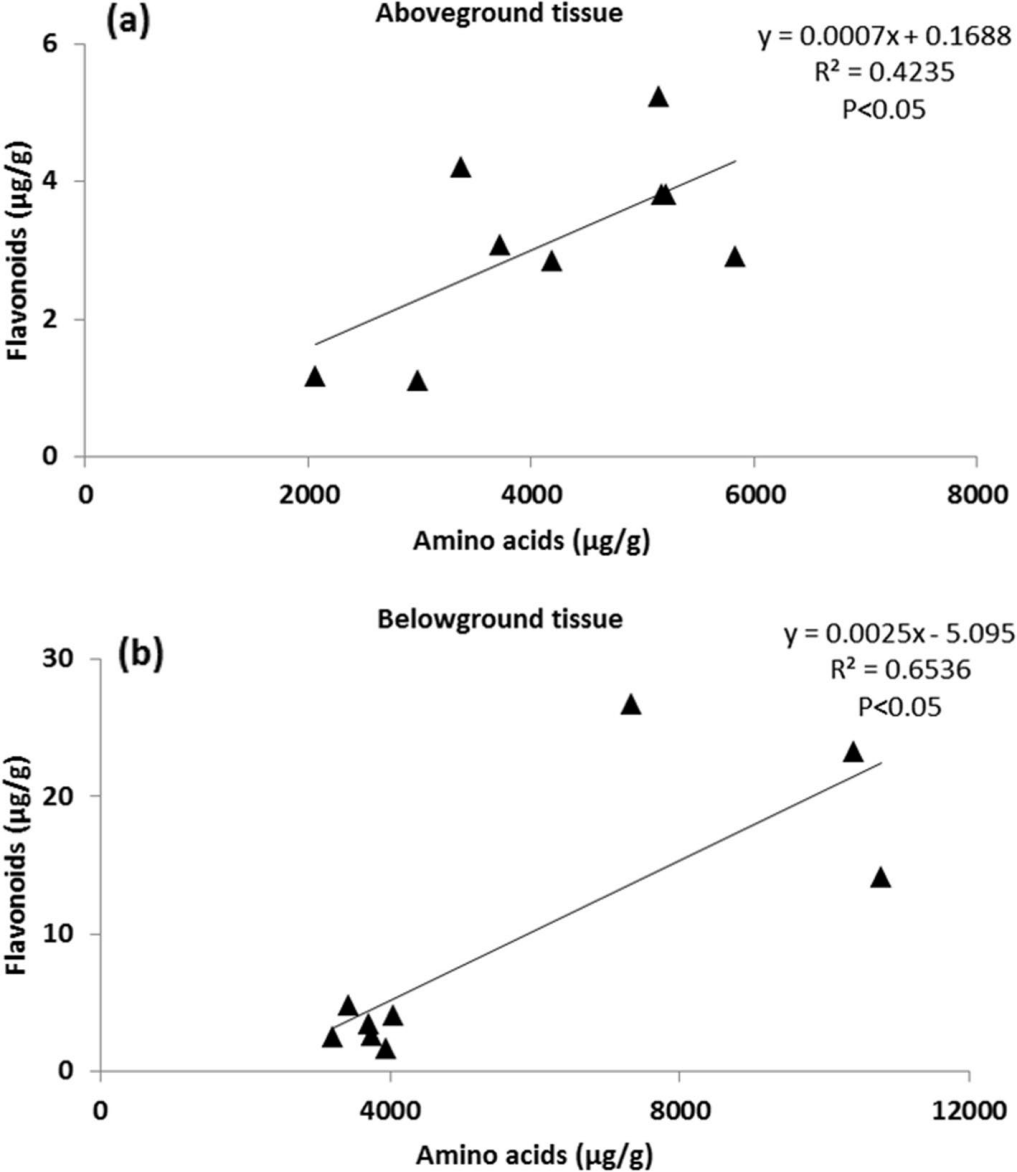
Relationship of amino acids and flavonoids in the aboveground (**a**) and belowground (**b**) tissues of *Thalassia hemprichii* under different sediment types

The relationships between amino acids and flavonoids in the above- and belowground tissues were significantly positive (Fig. 3). Meanwhile, linear regression tests were performed between the concentrations of total flavonoid and amino acid and sediment sand composition. The results showed that the amino acids in both above- and belowground tissues and total flavonoids in the belowground tissue were significantly negatively correlated with the sediment sand composition (Table 5).

**Table 5:** Correlation coefficients (r) and significance values (p) between the total flavonoids and amino acids concentration and sediment sand composition (grain size)

Parameters	r	*P*
Amino acids in aboveground tissue	−0.682	< 0.05
Amino acids in belowground tissue	−0.928	< 0.01
Flavonoids in aboveground tissue	−0.475	0.197
Flavonoids in belowground tissue	−0.933	< 0.01

### δ^34^S content

**Fig. 4:**
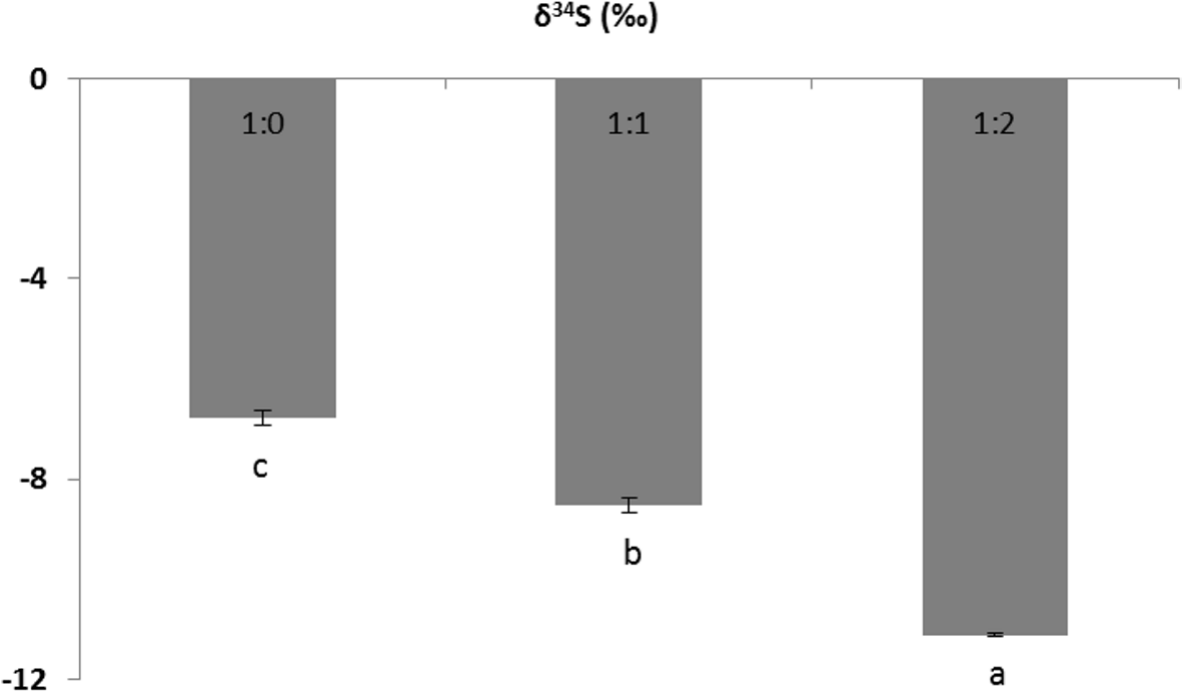
Effect of sediment type on the sulfur stable isotope (δ^34^S) in the belowground tissue of seagrass *Thalassia hemprichii*. Different letters on column indicate significant difference (*P* < 0.05). 1:0, the in-situ sediment without combining with coarse beach sand was added in the tank; 1:1, the combination of half in-situ sediment and half coarse beach sand was added in the tank; 1:2, the combination of 1/3 in-situ sediment and 2/3 coarse beach sand was added in the tank

The effects of sediment type on the δ^34^S content in the belowground tissue of *T. hemprichii* were depicted in Fig. 4. A significant difference was observed in the δ^34^S content, with higher values in the belowground tissue in the sediment with smaller particle sizes.

## Discussion

Decreased sediment particle size induced by increased inputs of fish food debris undoubtedly leads to anoxic conditions. Seagrass growth and survival may be constrained by anoxic sediment conditions. Anoxia is regarded as one of the most harmful factors for plants because of the accumulation of toxic end products [23]. Furthermore, sulphide toxicity is considered one of the main contributing factors to the global decline of seagrass beds [32]. Under such circumstances, seagrass photosynthesis would be directly affected and could regulate responses through changes in primary and secondary metabolites. To the best of our knowledge, this was the first report on the response of seagrass secondary metabolic to environmental stress using targeted metabolomics.

### Seagrass photosynthesis was indirectly enhanced by adding coarse beach sand

Seagrass-sediment interactions are dynamic [7]. The present results showed that Y (II) was not significantly different at either stage among the treatments, implying that this parameter was not a good indicator of stress. Meanwhile, there was almost little change in the parameters of the rapid light curve on day 6 among the treatments, indicating that this effect was not obvious at the initial stage. However, a noticeable enhancement in rETR_max_ and Ek_ETR_ was observed on day 21 (at the end of the experiment) under the coarse beach sand addition treatment, suggesting the ability to transfer more electrons and larger energy investment for CO_2_ fixation, scavenging reactive oxygen species [35], photosystem photoprotection [36], nitrogen assimilation and redox signalling pathways [37] in *T. hemprichii*. Similarly, a higher sand composition induced a higher Eh, indicating that coarse beach sand addition increased sediment porosity, therefore benefiting oxygen permeation. This favourable condition might benefit seagrass growth by enhancing photosynthesis. Conversely, higher photosynthesis also induced a positive effect on the sediment redox potential [38]. High photosynthesis resulted in greater pools of O_2_ in belowground tissue, enhancing the radial O_2_ loss and the oxic shield [39, 40]. Furthermore, O_2_ consumption by seagrass roots increased with increasing shoot-to-root mass ratio, which was dominated by root mass and disrupted by sulphide [41]. The effect of the sediment might show a specific difference. Some seagrass species such as *Zostera marina* and *Cymodocea nodosa* showed greater tolerance to reducing conditions in sediments than *T. hemprichii* [23]. Muddy sediments might be more favourable for *Z. marina* than sandy sediments, although they can be grown in either sediment [29]. Sediments with high silt and clay contents could promote the successful transplantation of *Z. marina* [42].

### Belowground amino acids and flavonoids were stimulated to counteract anoxic stress in sediment with smaller particle sizes

Amino acids are important for protein biosynthesis, other metabolic pathways, and signal transduction [43]. Proline and sarcosine were the main amino acids in both the above- and belowground tissues of *T. hemprichii*. Asparagic acid and proline were the main amino acids in both the above- and belowground tissues of *Posidonia oceanica* and *C. nodosa*, respectively [44]. Amino acids may change substantially in response to environmental factors. The present study indicated that the total amino acid content in the above- and belowground tissues were both higher in smaller sediment particle sizes. In particular, the belowground amino acid concentration in the smaller sediment particle sizes was more than twice that in the larger sediment particle sizes. This phenomenon could be attributed to two reasons. First, increased ammonium assimilation was induced by the higher nitrogen content in sediments with smaller particle sizes [22]. Second, the adverse effects of lower oxygen conditions. Higher alanine (an end product of anaerobic fermentation in higher plants) and proline contents in both above- and belowground tissues of *T. hemprichii* were observed in the smaller sediment particle size with lower Eh. Similarly, the alanine concentration was enhanced in *Z. marina* [12, 45] and *P. oceanica* [23] under anoxic condition. Alanine enhancement is a known phenomenon due to pyruvate accumulation in plants subjected to anoxia, which would mitigate cell acidification [21, 43] and provides support for carbon metabolism and energy homeostasis by linking glycolysis with the tricarboxylic acid cycle [46]. The increase in alanine occurs at the expense of glutamate and aspartate, and concomitantly with the GABA accumulation [47]. Furthermore, leucine and valine, the two branched-chain amino acids, were also enhanced, which could be synthesized de novo from pyruvate [43]. Proline in most higher plants often responds to an increase in concentration under environmental constraints, including salinity, drought, and anaerobiosis [43]. Increased proline content is also a factor in free radical detoxification in flooded corn plants [46]. Moreover, excess sulphate is also reduced to sulphide and incorporated into methionine, a sulphur-containing amino acid [48]. Significantly higher methionine in the belowground tissue was observed in smaller sediment particle sizes, indicating that methionine biosynthesis might function as a detoxification agent for excess sulphate or sulphide. Similarly, *Z. marina* also detoxified gaseous sediment-derived sulphide through incorporation, and most of the detoxification occurred in the belowground tissues, where sulphide intrusion was the greatest [49].

Among phenolic compounds, flavonoids are potentially reliable biomarkers of environmental quality [50]. The present study indicated that galuteolin and luteolin were the prime flavones in the aboveground tissue of *T. hemprichii*, whereas catechin and isoquercitrin were the main components in belowground tissue. In *P. oceanica*, myricetin and isorhamnetin were the main constituents of leaf flavonols [50]. The flavonoid of *Halophila stipulacea* was dominated by apigenin-7-O-β-glucopyranoside [51]. Seagrasses with larger leaves and/or more pairs of cross-veins in the leaves contained sulfated flavonoids, whereas those with smaller leaves and/or fewer cross-veins lacked these compounds [52]. This difference might be associated with the measurement method or specific differences. Low oxygen stress changed the expression of metabolic genes, such as flavonoid biosynthesis, and induced flavonoid biosynthesis that involves methylation as a modification of compounds to accomplish activation or intracellular translocation [53]. The present study showed that lower flavonoid concentrations in belowground tissue were observed in sediments with larger particle sizes. Similarly, a decrease in the total phenolic concentration in *Z. marina* was also observed when grown in high pCO_2_ waters [24]. This might be attributed to the reallocation of carbon to other pathways [54]. Phenolic compounds are regarded as storage compounds for carbohydrates, which are only produced when plants cannot convert carbohydrates into growth [50, 55]. Ecological plant strategy theory implies that plants investing in biochemical means of stress protection are likely to invest less carbon in constitutive productivity [31]. A trade-off mechanism between growth and secondary production for protection might occur in the present study, which required further research. Interestingly, the δ^34^S in the belowground tissue of *T. hemprichii* was more positive in the sediment with smaller particle size, which was similar to the change in belowground flavonoids. Flavonoids sulfation might facilitate the consumption of intruded sulphide, which functions as a detoxification agent [32]. *Z. marine* and *T. testudinum*, which are rich in flavonoid sulfates, could tolerate higher sulphide intrusion than *P. oceanica*, with an almost total absence of flavonoids [32, 56–59]. Fifty precent of the radiolabelled sulfate fed to *Z. marina* was recovered from the phenolic flavonoid fraction [60]. Flavonoid sulfates might play a key role in the allelochemical relationships of seagrasses [59, 61]. In particular, catechin was extremely higher in the belowground tissue of *T. hemprichii* in the sediment with smaller particle sizes. Catechin might play a crucial role in the response to anoxic conditions. Exogenous catechin can markedly reduce waterlogging injury in roots by sufficiently enhancing the free radical scavenging system to lower hydrogen peroxide and superoxide concentrations [62].

In the present study, a strong positive correlation between flavonoids and amino acids indicated that amino acids were a good indicator of flavonoid accumulation. The available aromatic amino acids are intended for the flavonoid pathway and provided by the primary metabolism [63], which was confirmed by the fact that aromatic amino acids including phenylalanine, tryptophan, and tyrosine, were higher in the in-situ sediment without combining with coarse beach sand. Leucine and valine are precursors of plant secondary metabolites. Further research is needed to perform a cross phytochemical/phylogenetic analysis of seagrasses to correlate the phenolic fingerprint and amino acid sequences of the genes encoding the flavonoid pathway [61].

### Ecological significance

**Fig. 5:**
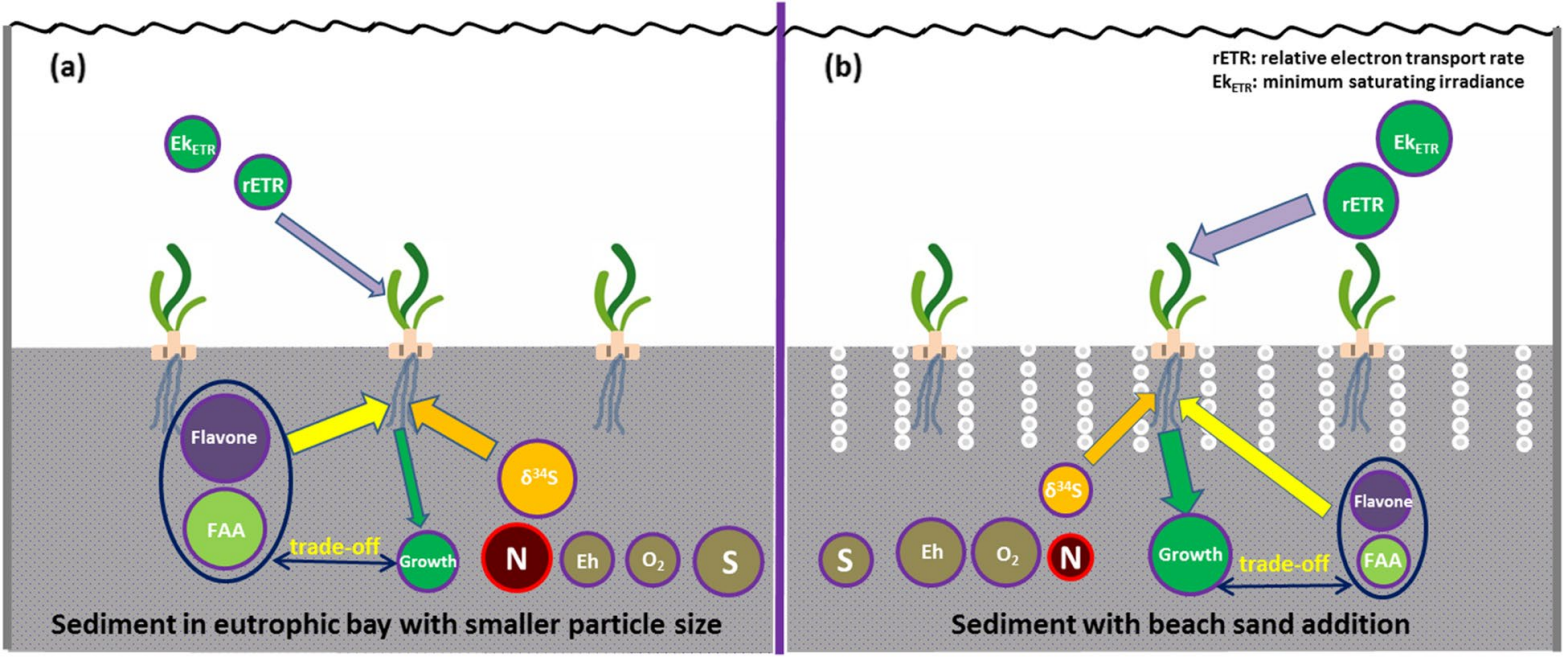
Schematic pictures of the effect of sediment on the photosynthesis, stable isotope sulfur (δ^34^S), FAA (free amino acid) and flavone of seagrasses. **a** indicated that seagrass growing in sediment in eutrophic bay with lower particle size, showed smaller rETR (relative electron transport rate) and Ek_ETR_ (the minimum saturating irradiance), while higher FAA and Flavone accumulated in the belowground tissue to counteract anoxic stress. **b** indicated that beach sand addition indirectly enhanced rETR and Ek_ETR_ by improving the growth condition for seagrass with lower flavone and FAA. N: sediment nitrogen. S: sediment sulfur. Eh: sediment redox potential, measuring the oxidation/reduction state. The bigger the circle, the higher the content or value

Sediment type is a key factor influencing seagrass growth and success rate of transplantation [7, 64]. Recently, modifications of sediment structure and composition by removing polluted sediment and adding exogenous matrices have often been applied to better protect submerged plants and ecological restoration projects of rivers and lakes [65–67]. However, sediment type modification has been less considered and applied in the ecological restoration of coastal zones, especially in seagrass beds suffering from eutrophication. Seagrass *T. hemprichii* in the sediment with smaller particle sizes exhibited lower rETR_max_ and Ek_ETR_, indicating a decrease in light tolerance (Fig. 5). Organic matter input from shrimp pond cultures along the Xincun bay coastline resulted in smaller sediment particle sizes. This induced that *T. hemprichii* in the high intertidal area suffered more from high light stress during air exposure, causing a faster decline in the high than lower intertidal area. The present study proved that adding coarse beach sand would reduce sediment total nitrogen, organic matter, and sulphur content and enhance oxygen permeability in the hypoxic/anoxic sediment, leading to less synthesis of amino acids and flavonoids. This would benefit seagrass photosynthesis and allocate more carbon to growth. The sediment particle sizes in the eutrophic area could also be modified into the same sediment of *T. hemprichii* growing in offshore and low-impact areas, with corresponding sand, silt, and clay compositions as (97.60 ± 1.70)%, (2.40 ± 1.70)%, and (0.00 ± 0.00)%, respectively [68]. Furthermore, stimulated photosynthesis also led to less toxic substance accumulation by increasing oxygenated conditions in the rhizosphere [38], and seagrasses do not have to transfer photosynthetic products, such as carbohydrates and secondary metabolites, to overcome the toxic effects of sulfide. This would benefit and accelerate seagrass growth. The enhancement of rETR_max_ and Ek_ETR_ may partially offset the negative effects of reduced light irradiance on C balance and improve high light tolerance. In particular, seagrass beds worldwide have faced increased eutrophication caused by a large input of nutrients from anthropogenic activity [4, 22, 69]. Considering the large variation of seagrass leaf light absorption [70, 71], the leaf light absorption needs to be measured. Field observations concerning seagrass response to sediment type by applying chlorophyll fluorescence and oxygen evolution [72–74], are needed at an ecosystem level to determine the operable habitat requirements of seagrasses [64]. It is also very important to change the sediment type to improve the growth conditions of seagrass and enhance the success rate of planting and transplanting seagrass shoots in eutrophic ecosystems. Cage farming and shrimp pond cultures in *T. hemprichii* beds should also be reduced or prohibited to decrease the input of food debris. Furthermore, *T. hemprichii* is gradually being replaced by *Enhalus acoroides* owing to a decrease in sediment particle size. Meanwhile, sediment type also affected interspecific competition between salt marsh plants [75]. Thus, further studies on the effect of changing sediment on interspecific competition and community succession in seagrasses are needed.

## Conclusion

Together, our results indicated that coarse beach sand addition could indirectly enhance the photosynthesis of *T. hemprichii* by improving sediment conditions with lower total nitrogen, organic matter and sulphide intrusion. Meanwhile, considerably greater belowground amino acids and flavonoids counteracted anoxic stress in sediments with smaller particle sizes, leading to more positive belowground δ^34^S. Consequently, the sediment could be modified in the eutrophic bay to improve the growth conditions for dominant tropical seagrass *T. hemprichii*. However, more detailed analyses and field experiments on the response of seagrass to different sediment types are required. Further studies are needed to examine the metabolic pathways of key primary and secondary metabolites of seagrass and trade-off mechanisms between growth and defence, under sediment modification.

## Material and methods

Approximately 250 intact shoots of healthy *T. hemprichii* (the identification was undertaken by Dr. Tan, and related voucher specimen was shown in Additional file 2) were collected in the same patch to avoid patch differences. It is a sand-clay site with a water depth of ~ 2 m in Xincun Bay (18°24′34″N -18°24′42″N, 109°57′42″E-109°57′58″E), located southeast of Hainan Island, Southern China (Additional file 3). The seagrass density was between 208 and 340 shoots/m^2^, and the biomass ratio of aboveground to belowground tissue was between 0.15 and 0.20. Plants were collected carefully to keep belowground structures intact and immediately transported to the laboratory in covered buckets containing seawater. Two boxes of in-situ sediment below *T. hemprichii* and one box of coarse beach sand without sieving from the coastline were also collected. Plants were gently washed with in-situ seawater, separated into single shoots, and then cultured in an aquarium with in-situ seawater and sediment for 7 d prior to the start of the experiments. The light intensity at the surface of the seagrass leaves was 150 μmol photons m^−2^ s^−1^, and the temperature was maintained at 25 °C using air conditioning. The light was applied with 400 W metal-halide lamps and was set on a 12 h cycle.

### Experimental design

**Fig. 6:**
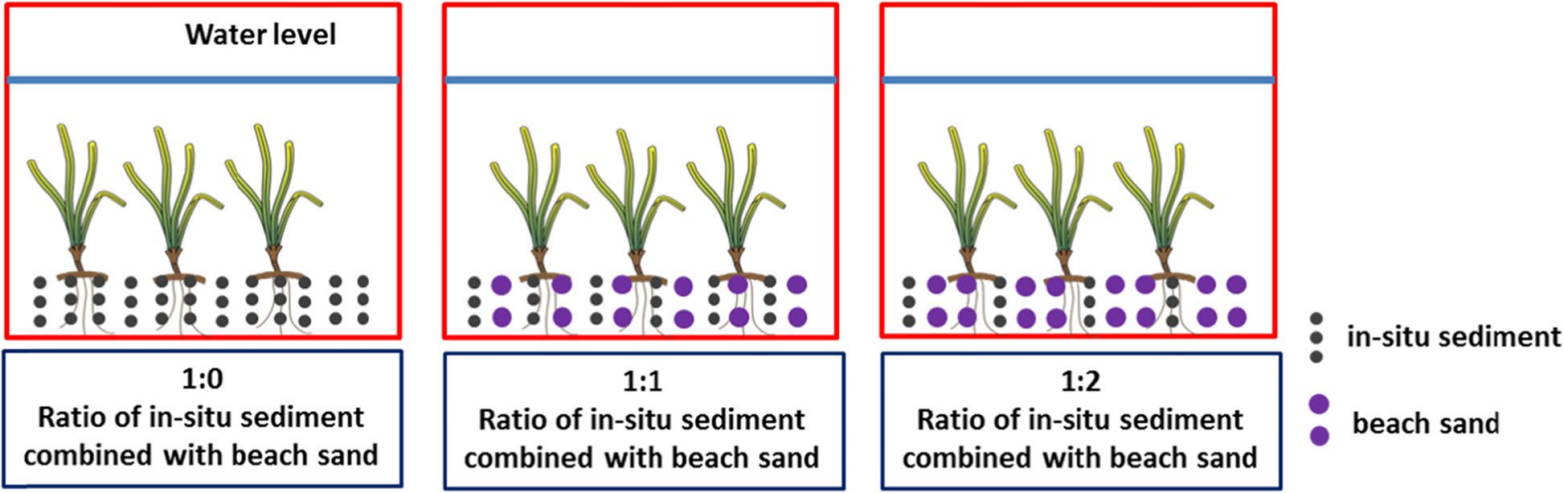
Experimental set-up of the laboratory treatment. 1:0, the in-situ sediment without combining with coarse beach sand was added in the tank; 1:1, the combination of half in-situ sediment and half coarse beach sand was added in the tank; 1:2, the combination of 1/3 in-situ sediment and 2/3 coarse beach sand was added in the tank

*T. hemprichii* was cultured in three sediment types with in-situ sediment combinations with different ratios of coarse beach sand. Each sediment type treatment had three replicates. There were nine glass tanks (270 × 220 × 250 mm) with 20 shoots in each tank (Fig. 6). The sediment thickness was 8 cm and the overlying seawater was 8.91 L. The seawater pH, salinity and dissolved inorganic nitrogen were 8.08 ± 0.04, 30.45 ± 0.92, 7.45 ± 0.74 μmol L^− 1^, respectively. Seawater was aerated without replicating tides, as the seagrasses collected were in the lower intertidal zone with little air exposure. 1:0 represented the in-situ sediment without combining with coarse beach sand added to the tank; 1:1 represented the combination of half (volume) in-situ sediment and a half (volume) coarse beach sand added to the tank; 1:2 represented the combination of 1/3 in-situ sediment and 2/3 coarse beach sand added to in the tank. The physiochemical parameters of the sediments were showed in Table 6. The concentrations of sediment organic carbon, total nitrogen, organic matter, and sulfur under the 1:0 treatment were higher than those of the other two treatments, whereas the δ^34^S value exhibited a contrasting trend. For the particle sizes, an increasing trend was observed for sand composition from 1:0, 1:1, and 1:2, whereas the clay composition showed an inverse trend (Table 6). For the sand composition, a decreasing trend was found for the coarse sand composition from 1:0, 1:1, and 1:2, whereas fine sand showed a contrasting trend. The plants were maintained under these conditions for 21 d.

**Table 6:** The sediment physiochemical parameters including pH (*n* = 9), nitrogen (*n* = 9), carbon (*n* = 9), organic matter (*n* = 9), sulfur (*n* = 9), δ^34^S (*n* = 9) and particle sizes (*n* = 3) in the initial stage of the three sediment types with in-situ sediment combination with different ratio of coarse beach send. 1:0, the in-situ sediment without combining with coarse beach sand was added in the tank; 1:1, the combination of half in-situ sediment and half coarse beach sand was added in the tank; 1:2, the combination of 1/3 in-situ sediment and 2/3 coarse beach sand was added in the tank

Sediment type	pH	N (%)	C (%)	Organic matter (%)	S (%)	δ^34^S (‰)	Particle sizes					
							Coarse sand (%)	Medium sand (%)	Fine sand (%)	Very fine sand (%)	Silt (%)	Clay (%)
1:0	8.83 ± 0.04^a^	0.034 ± 0.005^b^	0.344 ± 0.059^b^	1.38 ± 0.11^a^	0.017 ± 0.001^b^	−0.80 ± 0.49^a^	0.254	31.237	58.067	3.628	5.244	1.570
1:1	8.64 ± 0.02^b^	0.025 ± 0.002^a^	0.253 ± 0.029^ab^	1.11 ± 0.17^b^	0.013 ± 0.005^ab^	1.09 ± 1.14^ab^	6.274	44.017	41.076	3.842	3.369	1.422
1:2	8.92 ± 0.04^c^	0.020 ± 0.003^a^	0.216 ± 0.045^a^	0.90 ± 0.03^b^	0.009 ± 0.001^a^	2.60 ± 1.19^b^	11.185	44.667	35.584	4.149	3.842	0.573

The different lower case letters indicated significant differences among treatments

### Photosynthetic performance and biochemical analysis

A PAM fluorometer (Mini-PAM, WALZ GmbH) was used to generate effective quantum yield (Y (II)) and rapid light curves (RLCs). Y (II) measured in light-adapted leaves indicates the amount of energy used in photochemistry [76]. Photosynthetic performance was measured in the same shoots in each tank on days 6 and day 21. Each sediment type treatment had three replicates of RLC. Y (II) was measured after the application of a saturating pulse of light (measuring intensity of < 0.15 μmol photons m^− 2^ s^− 1^, saturating intensity of > 4000 μmol photons m^− 2^ s^− 1^, saturation width of 0.8 s). In the absence of dark acclimation, an effective quantum yield measurement was taken at the beginning of each rapid light curve, before the actinic light from the Mini-PAM, and at the end of each 10 s irradiance step, resulting in nine effective quantum yield measurements for each RLC [76]. The illumination time for RLCs might be short. This type of determination cannot be used to estimate the equivalent descriptors derived from the classic photosynthetic response curve to irradiance. However, the present study took standardized measurements on the RLCs, which might provide useful information for the description of relative changes in photosynthetic activity [73, 74, 77]. The process of measuring RLCs and the determination of the relative maximum electron transport rate (rETR_max_), minimum saturating irradiance (Ek_ETR_) and α_ETR_ (the initial slope of the light-limited relationship) by curve-fitting were according to Ralph and Gademann [76] and Jiang et al. [78].

At the end of the experiment, the plants were carefully retrieved and separated into above- and belowground tissues. Subsamples were oven-dried (60 °C) and individually powdered with a grinder to pass through an 80-mesh sieve (with a mesh diameter of 0.18 mm) for measuring nutrients and stable sulfur isotopes, whereas the other subsamples were sent for measuring compositions of free amino acids and flavonoids with dry ice. The concentration of tissue nitrogen was determined using a CHN analyzer (Elementar, Vario EL-III, Germany). The stable isotope sulfur and sulfur contents were measured with a DELTA V Advantage isotope mass spectrometer and an EA-HT elemental analyzer. Amino acids were measured using Waters Quattro Premier XE, whereas flavonoids were measured using Waters ACQUITY UPLC and Triple quadrupole mass spectrometer (AB 4000).

### Sediment analysis

The particle sizes of the sediment samples, divided into three groups (< 4 μm (clay), 4–63 μm (silt), and > 63 μm (sand)), were analyzed using a laser diffractometer (Malvem Mastersizer 2000) [79]. Sediment samples were processed according to Jiang et al. [26] before measuring sediment organic carbon and total nitrogen concentrations using a CHN analyzer (Elementar, Vario EL-III, Germany). Sediment organic matter content was analyzed by sediment calcination in a muffle furnace (550 °C for 4 h) [80]. Sediment pH was measured in distilled water with a 1:2.5 sediment/solution ratio using a portable pH acidometer (PHB-4).

At the end of the experiment, sediment redox potential (Eh, measuring the oxidation/reduction state) was measured using an oxidation-reduction potentiometer (Mettler Toledo, Seven 2 Go).

### Statistical analysis

The means and standard errors of all variables were calculated, and all data were first tested to determine whether the assumptions of homogeneity of variance and normality were met. Where these assumptions were not met, the raw data were transformed, and further statistical analysis was conducted using the dataset that fulfilled the assumptions. The effect of sediment type was analyzed by one-way analysis of variance using SPSS for Windows version 18. Treatment means were compared and separated using the least significant difference at *P* < 0.05. A multiple comparison test that did not assume equal variances was Dunnett’s T3 (Additional files 4, 5, 6).

## Supplementary Information

**Additional file 1: Figure S1.** Change trend of Y(II) (effective quantum yield).

**Additional file 2: Figure S2.** Voucher specimen of *Thalassia hemprichii.*

**Additional file 3: Figure S3.** The *Thalassia hemprichii* bed in Xincun Bay.

**Additional file 4: Table S1.** Results of Levene’s test of homogeneity of photosynthesis and nutrient.

**Additional file 5: Table S2.** Results of Levene’s test of homogeneity of amino acids.

**Additional file 6: Table S3.** Results of Levene’s test of homogeneity of flavonoid.

## Data Availability

The data generated or analyzed in this study are included in this article and its supplementary information files. Other materials that support the findings of this study are available from the corresponding author on reasonable request.

## Abbreviations

rETR_max_: Relative maximum electron transport rate
Ek_ETR_: Minimum saturating irradiance
α_ETR_: The initial slope of the light-limited relationship
1:0: The in-situ sediment without coarse beach sand was added to the tank
1:1: The combination of half in-situ sediment and half coarse beach sand was added to the tank
1:2: The combination of 1/3 in-situ sediment and 2/3 coarse beach sand was added to the tank

## References

[1] Hemminga M, Duarte CM (2000). Seagrass ecology.

[2] Larkum AW, Orth RRJ, Duarte CM (2006). Seagrasses: biology, ecology, and conservation.

[3] Mohr W, Lehnen N, Ahmerkamp S, Marchant HK, Graf JS, Tschitschko B, Yilmaz P, Littmann S, Gruber-Vodicka H, Leisch N (2021). Terrestrial-type nitrogen-fixing symbiosis between seagrass and a marine bacterium. Nature.

[4] Jiang Z, Liu S, Zhang J, Wu Y, Zhao C, Lian Z, Huang X (2018). Eutrophication indirectly reduced carbon sequestration in a tropical seagrass bed. Plant Soil.

[5] Waycott M, Duarte CM, Carruthers TJ, Orth RJ, Dennison WC, Olyarnik S, Calladine A, Fourqurean JW, Heck KL, Hughes AR (2009). Accelerating loss of seagrasses across the globe threatens coastal ecosystems. Proc Natl Acad Sci U S A.

[6] Burkholder JM, Tomasko DA, Touchette BW (2007). Seagrasses and eutrophication. J Exp Mar Biol Ecol.

[7] de Boer WF (2007). Seagrass–sediment interactions, positive feedbacks and critical thresholds for occurrence: a review. Hydrobiologia.

[8] Bishop MJ, Kelaher BP (2013). Replacement of native seagrass with invasive algal detritus: impacts to estuarine sediment communities. Biol Invasions.

[9] Liu S, Jiang Z, Zhang J, Wu Y, Lian Z, Huang X (2016). Effect of nutrient enrichment on the source and composition of sediment organic carbon in tropical seagrass beds in the South China Sea. Mar Pollut Bull.

[10] Holmer M, Bondgaard EJ (2001). Photosynthetic and growth response of eelgrass to low oxygen and high sulfide concentrations during hypoxic events. Aquat Bot.

[11] Pregnall A, Smith R, Kursar T, Alberte R (1984). Metabolic adaptation of Zostera marina (eelgrass) to diurnal periods of root anoxia. Mar Biol.

[12] Pregnall A (2004). Effects of aerobic versus anoxic conditions on glutamine synthetase activity in eelgrass (*Zostera marina* L.) roots: regulation of ammonium assimilation potential. J Exp Mar Biol Ecol.

[13] Govers LL, de Brouwer JHF, Suykerbuyk W, Bouma TJ, Lamers LPM, Smolders AJP, van Katwijk MM (2014). Toxic effects of increased sediment nutrient and organic matter loading on the seagrass Zostera noltii. Aquat Toxicol.

[14] Holmer M, Hasler-Sheetal H. Sulfide intrusion in seagrasses assessed by stable sulfur isotopes—a synthesis of current results. Front Mar Sci. 2014;1(64). 10.3389/fmars.2014.00064).

[15] Erskine JM, Koch MS (2000). Sulfide effects on *Thalassia testudinum* carbon balance and adenylate energy charge. Aquat Bot.

[16] Macreadie PI, Schliep MT, Rasheed MA, Chartrand KM, Ralph PJ (2014). Molecular indicators of chronic seagrass stress: a new era in the management of seagrass ecosystems?. Ecol Indic.

[17] Orth RJ, Carruthers TJ, Dennison WC, Duarte CM, Fourqurean JW, Heck KL, Hughes AR, Kendrick GA, Kenworthy WJ, Olyarnik S (2006). A global crisis for seagrass ecosystems. Bioscience.

[18] Kumar M, Ralph P (2017). Systems biology of marine ecosystems.

[19] Kumar M, Kuzhiumparambil U, Pernice M, Jiang Z, Ralph PJ (2016). Metabolomics: an emerging frontier of systems biology in marine macrophytes. Algal Res.

[20] Hammer KJ, Borum J, Hasler-Sheetal H, Shields EC, Sand-Jensen K, Moore KA (2018). High temperatures cause reduced growth, plant death and metabolic changes in eelgrass Zostera marina. Mar Ecol Prog Ser.

[21] Hasler-Sheetal H, Fragner L, Holmer M, Weckwerth W (2015). Diurnal effects of anoxia on the metabolome of the seagrass *Zostera marina*. Metabolomics.

[22] de Kock W, Hasler-Sheetal H, Holmer M, Tsapakis M, Apostolaki ET (2020). Metabolomics and traditional indicators unveil stress of a seagrass (*Cymodocea nodosa*) meadow at intermediate distance from a fish farm. Ecol Indic.

[23] Pérez M, Invers O, Ruiz JM, Frederiksen MS, Holmer M (2007). Physiological responses of the seagrass *Posidonia oceanica* to elevated organic matter content in sediments: An experimental assessment. J Exp Mar Biol Ecol.

[24] Arnold T, Freundlich G, Weilnau T, Verdi A, Tibbetts IR (2014). Impacts of groundwater discharge at myora springs (north Stradbroke Island, Australia) on the phenolic metabolism of eelgrass, *Zostera muelleri*, and grazing by the juvenile rabbitfish, *Siganus fuscescens*. Plos One.

[25] Chiu S-H, Huang Y-H, Lin H-J (2013). Carbon budget of leaves of the tropical intertidal seagrass *Thalassia hemprichii*. Estuar Coast Shelf Sci.

[26] Jiang Z, Liu S, Zhang J, Zhao C, Wu Y, Yu S, Xia Z, Chi H, Huang X, Kumar M (2017). Newly discovered seagrass beds and their potential for blue carbon in the coastal seas of Hainan Island, South China Sea. Mar Pollut Bull.

[27] Li Q, Huang W, Zhou Y (2010). A preliminary study of eutrophication and occurrence of red tides in Xincun harbour. T Oceanol Limnol.

[28] Huang X, Jiang Z, Liu S, Yu S, Wu Y, Zhang J (2019). Study on ecology of tropical seagrass in China.

[29] van Katwijk MM, Wijgergangs LJM (2004). Effects of locally varying exposure, sediment type and low-tide water cover on *Zostera marina* recruitment from seed. Aquat Bot.

[30] Hofmann RW, Jahufer MZ (2011). Tradeoff between biomass and flavonoid accumulation in white clover reflects contrasting plant strategies. Plos One.

[31] Grime J (2001). Plant strategies, vegetation processes, and ecosystem properties.

[32] Grignon-Dubois M, Rezzonico B (2018). Phenolic chemistry of the seagrass *Zostera noltei* Hornem. Part 1: first evidence of three infraspecific flavonoid chemotypes in three distinctive geographical regions. Phytochemistry.

[33] Beer S, Björk M (2000). Measuring rates of photosynthesis of two tropical seagrasses by pulse amplitude modulated (PAM) fluorometry. Aquat Bot.

[34] Ralph PJ, Gademann R, Dennison WC (1998). In situ seagrass photosynthesis measured using a submersible, pulse-amplitude modulated fluorometer. Mar Biol.

[35] Ivanov B, Khorobrykh S (2003). Participation of photosynthetic electron transport in production and scavenging of reactive oxygen species. Antioxid Redox Signal.

[36] Murchie EH, Niyogi KK (2011). Manipulation of photoprotection to improve plant photosynthesis. Plant Physiol.

[37] Hanke GT, Endo T, Satoh F, Hase T (2008). Altered photosynthetic electron channelling into cyclic electron flow and nitrite assimilation in a mutant of ferredoxin: NADP (H) reductase. Plant Cell Environ.

[38] Marbà N, Duarte CM, Terrados J, Halun Z, Gacia E, Fortes MD (2010). Effects of seagrass rhizospheres on sediment redox conditions in SE Asian coastal ecosystems. Estuar Coast.

[39] Koch MS, Erskine JM (2001). Sulfide as a phytotoxin to the tropical seagrass Thalassia testudinum: interactions with light, salinity and temperature. J Exp Mar Biol Ecol.

[40] Oakes JM, Connolly RM (2004). Causes of sulfur isotope variability in the seagrass, Zostera capricorni. J Exp Mar Biol Ecol.

[41] Peyer SM, Maricle BR, Young DR (2020). Effect of sulfide and the role of root mass on metabolic fluxes in the seagrass Zostera marina. Environ Exp Bot.

[42] Zhang Q, Liu J, Zhang P-D, Liu Y-S, Xu Q (2015). Effect of silt and clay percentage in sediment on the survival and growth of eelgrass *Zostera marina*: transplantation experiment in swan Lake on the eastern coast of Shandong peninsula, China. Aquat Bot.

[43] D'Mello JF (2015). Amino acids in higher plants.

[44] Pirc H, Wollenweber B (1988). Seasonal changes in nitrogen, free amino acids, and C/N ratio in Mediterranean seagrasses. Mar Ecol.

[45] Bailey-Serres J, Fukao T, Gibbs DJ, Holdsworth MJ, Lee SC, Licausi F, Perata P, Voesenek LA, van Dongen JT (2012). Making sense of low oxygen sensing. Trends Plant Sci.

[46] Yordanova RY, Popova LP (2007). Flooding-induced changes in photosynthesis and oxidative status in maize plants. Acta Physiol Plant.

[47] Good AG, Muench DG (1993). Long-term anaerobic metabolism in root tissue (metabolic products of pyruvate metabolism). Plant Physiol.

[48] Nikiforova V, Bielecka M, Gakiere B, Krueger S, Rinder J, Kempa S, Morcuende R, Scheible W-R, Hesse H, Hoefgen R (2006). Effect of sulfur availability on the integrity of amino acid biosynthesis in plants. Amino Acids.

[49] Hasler-Sheetal H, Holmer M (2015). Sulfide intrusion and detoxification in the seagrass Zostera marina. Plos One.

[50] Cannac M, Ferrat L, Pergent-Martini C, Pergent G, Pasqualini V (2006). Effects of fish farming on flavonoids in *Posidonia oceanica*. Sci Total Environ.

[51] Bitam F, Ciavatta ML, Carbone M, Manzo E, Mollo E, Gavagnin M (2010). Chemical analysis of flavonoid constituents of the seagrass *Halophila stipulacea*: first finding of malonylated derivatives in marine phanerogams. Biochem Syst Ecol.

[52] McMillan C (1986). Sulfated flavonoids and leaf morphology in the *Halophila ovalis*—H. minor complex (Hydrocharitaceae) of the indo-Pacific Ocean. Aquat Bot.

[53] Klok EJ, Wilson IW, Wilson D, Chapman SC, Ewing RM, Somerville SC, Peacock WJ, Dolferus R, Dennis ES (2002). Expression profile analysis of the low-oxygen response in Arabidopsis root cultures. Plant Cell.

[54] Groner ML, Burge CA, Cox R, Rivlin ND, Turner M, Van Alstyne KL, Wyllie-Echeverria S, Bucci J, Staudigel P, Friedman CS (2018). Oysters and eelgrass: potential partners in a high pCO_2_ ocean. Ecology.

[55] Waterman PG, Ross JA, Mckey DB (1984). Factors affecting levels of some phenolic compounds, digestibility, and nitrogen content of the mature leaves of *Barteria fistulosa* (Passifloraceae). J Chem Ecol.

[56] Grignon-Dubois M, Rezzonico B (2015). Phenolic fingerprint of the seagrass *Posidonia oceanica* from four locations in the Mediterranean Sea: first evidence for the large predominance of chicoric acid. Bot Mar.

[57] Rowley DC, Hansen MS, Rhodes D, Sotriffer CA, Ni H, McCammon JA, Bushman FD, Fenical W (2002). Thalassiolins A–C: new marine-derived inhibitors of HIV cDNA integrase. Bioorgan Med Chem.

[58] Harborne JB (1975). Flavonoid sulphates: a new class of Sulphur compounds in higher plants. Phytochemistry.

[59] McMillan C, Zapata O, Escobar L (1980). Sulphated phenolic compounds in seagrasses. Aquat Bot.

[60] Nissen P, Benson AA (1964). Absence of selenate esters and “selenolipid” in plants. Biochim Biophys Acta.

[61] Grignon-Dubois M, Rezzonico B (2012). First phytochemical evidence of chemotypes for the seagrass *Zostera noltii*. Plants.

[62] Yiu J-C, Tseng M-J, Liu C-W (2011). Exogenous catechin increases antioxidant enzyme activity and promotes flooding tolerance in tomato (*Solanum lycopersicum* L.). Plant Soil.

[63] Trantas EA, Koffas MAG, Xu P, Ververidis F. When plants produce not enough or at all: metabolic engineering of flavonoids in microbial hosts. Front Plant Sci. 2015;6:7. 10.3389/fpls.2015.00007. 10.3389/fpls.2015.00007 PMC4310283 25688249

[64] Livingston RJ, McGlynn SE, Niu X (1998). Factors controlling seagrass growth in a gulf coastal system: water and sediment quality and light. Aquat Bot.

[65] Li F, Qin Y, Zhu L, Xie Y, Liang S, Hu C, Chen X, Deng Z (2016). Effects of fragment size and sediment heterogeneity on the colonization and growth of Myriophyllum spicatum. Ecol Eng.

[66] Liu L, Xiang-Qi B, Wan J-Y, Dong B-C, Luo F-L, Li H-L, Fei-Hai Y (2016). Impacts of sediment type on the performance and composition of submerged macrophyte communities. Aquat Ecol.

[67] Smart JWBM (1986). Sediment-related mechanisms of growth limitation in submersed macrophytes. Ecology.

[68] Jiang Z, Zhao C, Yu S, Liu S, Cui L, Wu Y, Fang Y, Huang X (2019). Contrasting root length, nutrient content and carbon sequestration of seagrass growing in offshore carbonate and onshore terrigenous sediments in the South China Sea. Sci Total Environ.

[69] Marbà N, Díaz-Almela E, Duarte CM (2014). Mediterranean seagrass (*Posidonia oceanica*) loss between 1842 and 2009. Biol Conserv.

[70] Cayabyab NM, Enríquez S (2007). Leaf photoacclimatory responses of the tropical seagrass Thalassia testudinum under mesocosm conditions: a mechanistic scaling-up study. New Phytol.

[71] Enríquez S, Agustí S, Duarte CM (1994). Light absorption by marine macrophytes. Oecologia.

[72] Carr H, Björk M (2010). A methodological comparison of photosynthetic oxygen evolution and estimated electron transport rate in tropical ULVA (Chlorophyceae) species under different light and inorganic carbon conditions. J Phycol.

[73] González-Guerrero LA, Vásquez-Elizondo RM, López-Londoño T, Hernán G, Iglesias-Prieto R, Enríquez S. Validation of parameters and protocols derived from chlorophyll a fluorescence commonly utilised in marine ecophysiological studies. Funct Plant Biol. 2022;49:517–32. 10.1071/FP21101 34372966

[74] Silva J, Sharon Y, Santos R, Beer S (2009). Measuring seagrass photosynthesis: methods and applications. Aquat Biol.

[75] Li HL, Wang YY, An SQ, Zhi YB, Lei GC, Zhang MX (2014). Sediment type affects competition between a native and an exotic species in coastal China. Sci Rep.

[76] Ralph PJ, Gademann R (2005). Rapid light curves: a powerful tool to assess photosynthetic activity. Aquat Bot.

[77] Enríquez S, Borowitzka MA (2010). The use of the fluorescence signal in studies of seagrasses and macroalgae. Chlorophyll a fluorescence in aquatic sciences: methods and applications.

[78] Jiang ZJ, Huang XP, Zhang JP (2010). Effects of CO_2_ enrichment on photosynthesis, growth, and biochemical composition of seagrass *Thalassia hemprichii* (Ehrenb.) Aschers. J Integr Plant Biol.

[79] Folk RL, Andrews P, Lewis DW (1970). Detrital sedimentary rock classification and nomenclature for use in New Zealand. N Z J Geol Geophys.

[80] Heiri O, Lotter AF, Lemcke G (2001). Loss on ignition as a method for estimating organic and carbonate content in sediments: reproducibility and comparability of results. J Paleolimnol.

