# Supplementary material for: Sand supplementation favors tropical seagrass Thalassia hemprichii in eutrophic bay: implications for seagrass restoration and management
Source: BMC Plant Biol. 2022 Jun 16;22:296. doi: 10.1186/s12870-022-03647-0 (PMC9205049; doi:10.1186/s12870-022-03647-0)
Supplement: Supplementary file 1 — Additional file 1: Figure S1. Change trend of Y(II) (effective quantum yield). [file 12870_2022_3647_MOESM1_ESM.docx]

**Figure S1 Change trend of Y(II) (effective quantum yield)**
