# Supplementary figures and images for: Sand supplementation favors tropical seagrass Thalassia hemprichii in eutrophic bay: implications for seagrass restoration and management
Source: BMC Plant Biol. 2022 Jun 16;22:296. doi: 10.1186/s12870-022-03647-0 (PMC9205049; doi:10.1186/s12870-022-03647-0)

**Figure S2 Voucher specimen of *Thalassia hemprichii***

**
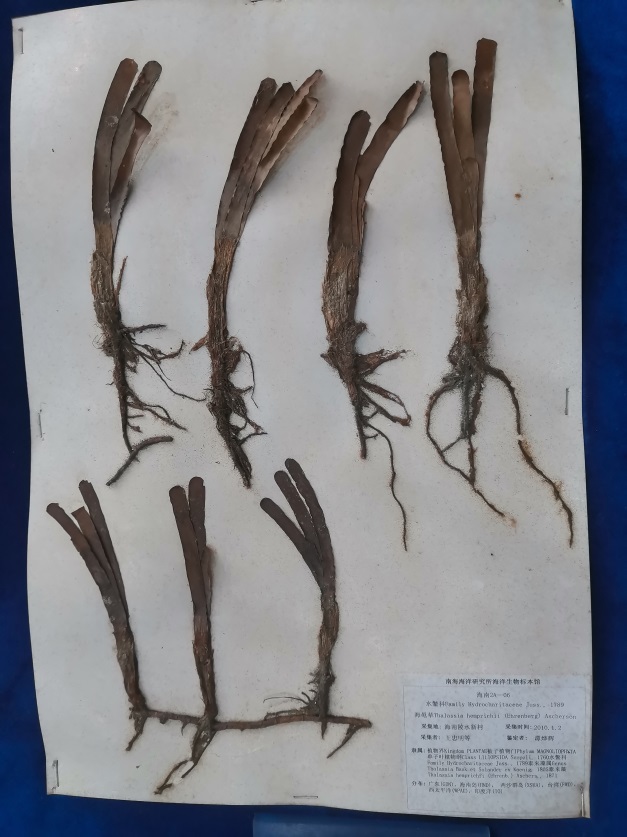
**

Supplement: Supplementary file 2 — Additional file 2: Figure S2. Voucher specimen of Thalassia hemprichii. [file 12870_2022_3647_MOESM2_ESM.docx]

**Figure S3 The *Thalassia hemprichii* bed in Xincun Bay**


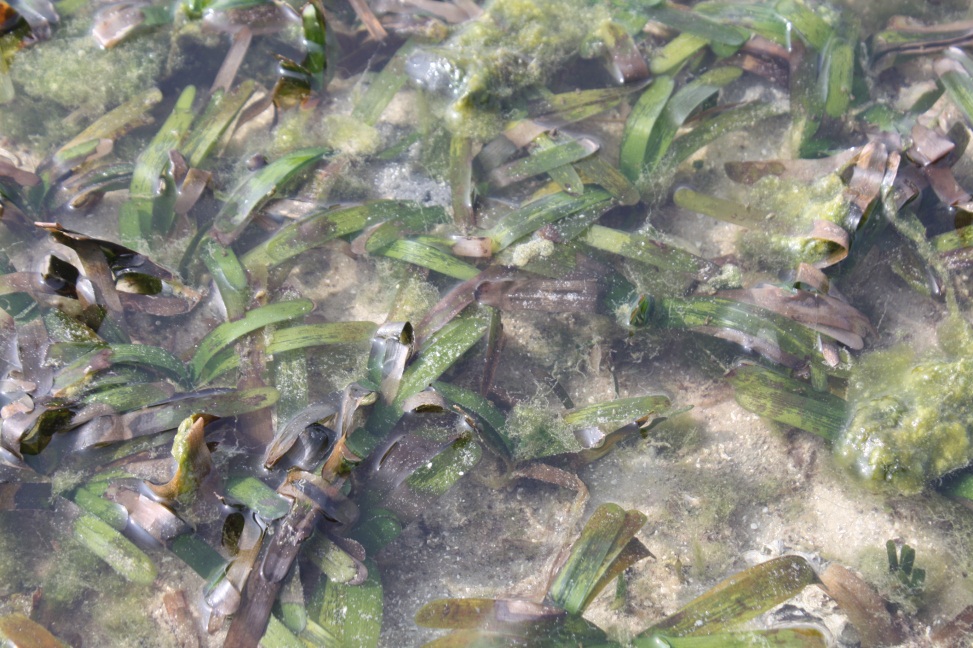

Supplement: Supplementary file 3 — Additional file 3: Figure S3. The Thalassia hemprichii bed in Xincun Bay. [file 12870_2022_3647_MOESM3_ESM.docx]
