# Supplementary material for: Sand supplementation favors tropical seagrass Thalassia hemprichii in eutrophic bay: implications for seagrass restoration and management
Source: BMC Plant Biol. 2022 Jun 16;22:296. doi: 10.1186/s12870-022-03647-0 (PMC9205049; doi:10.1186/s12870-022-03647-0)
Supplement: Supplementary file 4 — Additional file 4: Table S1. Results of Levene’s test of homogeneity of photosynthesis and nutrient. [file 12870_2022_3647_MOESM4_ESM.docx]

**Table S1** Results of Levene’s test of homogeneity of photosynthesis and nutrient

| Parameters | Levene Statistic | Sig. | Parameters | Levene Statistic | Sig. |
| --- | --- | --- | --- | --- | --- |
| Day 6 |  |  | Day 21 |  |  |
| Y(II) | 0.318 | 0.739 | Y(II) | 1.307 | 0.338 |
| rETR_max_ | 0.660 | 0.551 | rETR_max_ | 3.198 | 0.113 |
| α_ETR_ | 0.142 | 0.870 | α_ETR_ | 2.509 | 0.162 |
| Ek_ETR_ | 1.963 | 0.221 | Ek_ETR_ | 1.891 | 0.231 |
| leaf nitrogen | 0.618 | 0.570 | ratio of amino acid to leaf nitrogen | 1.067 | 0.401 |
