# Supplementary material for: Sand supplementation favors tropical seagrass Thalassia hemprichii in eutrophic bay: implications for seagrass restoration and management
Source: BMC Plant Biol. 2022 Jun 16;22:296. doi: 10.1186/s12870-022-03647-0 (PMC9205049; doi:10.1186/s12870-022-03647-0)
Supplement: Supplementary file 5 — Additional file 5: Table S2. Results of Levene’s test of homogeneity of amino acids. [file 12870_2022_3647_MOESM5_ESM.docx]

**Table S2** Results of Levene’s test of homogeneity of amino acids

| Parameters | Aboveground tissue | | Belowground tissue | | | |
| --- | --- | --- | --- | --- | --- | --- |
|  | Raw data | | Raw data | | Data transfer | |
|  | Levene Statistic | Sig. | Levene Statistic | Sig. | Levene Statistic | Sig. |
| Glycine | 0.267 | 0.774 | 11.213 | 0.009 | 3.504 | 0.098 |
| Sarcosine | 0.969 | 0.432 | 7.648 | 0.022 | 3.994 | 0.081 |
| Alanine | 2.835 | 0.136 | 4.945 | 0.054 |  |  |
| Valine | 0.439 | 0.664 | 2.058 | 0.209 |  |  |
| Proline | 2.423 | 0.169 | 6.531 | 0.031 | 3.071 | 0.121 |
| Threonine | 1.494 | 0.297 | 6.548 | 0.031 | 0.734 | 0.519 |
| Isoleucine | 0.563 | 0.597 | 9.149 | 0.015 | 1.185 | 0.368 |
| Leucine | 2.943 | 0.129 | 5.246 | 0.048 | 0.394 | 0.691 |
| Ornithine | 2.545 | 0.158 | 1.850 | 0.237 |  |  |
| Methionie | 2.936 | 0.129 | 2.584 | 0.155 |  |  |
| Histidine | 2.999 | 0.125 | 4.111 | 0.075 |  |  |
| Phenylalanine | 1.896 | 0.230 | 7.781 | 0.022 | 1.308 | 0.338 |
| Arginine | 2.724 | 0.144 | 1.815 | 0.242 |  |  |
| Tyrosine | 1.169 | 0.373 | 1.952 | 0.222 |  |  |
| Asparagic acid | 3.786 | 0.086 | 1.968 | 0.220 |  |  |
| Tryptophan | 1.805 | 0.243 | 5.313 | 0.047 | 3.376 | 0.104 |
| 4-aminobutyric acid | 2.996 | 0.125 | 2.780 | 0.140 |  |  |
| Serine | 1.538 | 0.289 | 8.966 | 0.016 | 0.373 | 0.704 |
| Lysine | 1.810 | 0.243 | 4.873 | 0.055 |  |  |
| Glutamate | 1.601 | 0.277 | 4.534 | 0.063 |  |  |
| Amino acid | 1.346 | 0.329 | 11.439 | 0.009 | 1.445 | 0.307 |
