# Supplementary material for: Sand supplementation favors tropical seagrass Thalassia hemprichii in eutrophic bay: implications for seagrass restoration and management
Source: BMC Plant Biol. 2022 Jun 16;22:296. doi: 10.1186/s12870-022-03647-0 (PMC9205049; doi:10.1186/s12870-022-03647-0)
Supplement: Supplementary file 6 — Additional file 6: Table S3. Results of Levene’s test of homogeneity of flavonoid. [file 12870_2022_3647_MOESM6_ESM.docx]

**Table S3** Results of Levene’s test of homogeneity of flavonoid

| composition | Aboveground tissue |  |  |  | Belowground tissue |  |  |  |
| --- | --- | --- | --- | --- | --- | --- | --- | --- |
|  | Raw data |  | Data transfer |  | Raw data |  | Ln transfer |  |
|  | Levene Statistic | Sig. | Levene Statistic | Sig. | Levene Statistic | Sig. | Levene Statistic | Sig. |
| Catechin | 1.739 | 0.254 |  |  | 7.424 | 0.024^+^ |  |  |
| Epicatechin | - |  |  |  | 15.725 | 0.004^+^ |  |  |
| Taxifolin | - |  |  |  | 0.072 | 0.931 |  |  |
| Galuteolin | 0.323 | 0.736 |  |  | 1.326 | 0.333 |  |  |
| Rutin | 3.960 | 0.080 |  |  | 1.759 | 0.250 |  |  |
| Isoquercitrin | 3.527 | 0.097 |  |  | 1.246 | 0.353 |  |  |
| Astragalin | - |  |  |  | 0.212 | 0.815 |  |  |
| Diosmin | 0.225 | 0.805 |  |  |  |  |  |  |
| Quercetin | 1.801 | 0.244 |  |  | 6.930 | 0.028 | 2.770 | 0.141 |
| Naringenin | 3.438 | 0.101 |  |  | 3.047 | 0.122 |  |  |
| Luteolin | 0.442 | 0.662 |  |  | 3.501 | 0.098 |  |  |
| Apigenin | 0.761 | 0.507 |  |  | 6.470 | 0.032 | 1.325 | 0.334 |
| Chrysin | 1.548 | 0.287 |  |  |  |  |  |  |
| Kaempferide | 8.892 | 0.016 | 4.928 | 0.054 |  |  |  |  |
| Total flavonoids | 0.098 | 0.908 |  |  | 5.814 | 0.039 | 0.139 | 0.873 |

+: Multiple comparison test that didn’t assume equal variances was Dunnett’s T3.
